# A Place to Which Ambulant Patients Come

**Published:** 1918-11-02

**Authors:** 


### THE DISPENSARY IN THE UNITED STATES.

### "A Place to which Ambulant Patients come."

The dispensary in the United States of America takes a more important part in public medical work than it does with us. There, the best dispensaries combine the work of the dispensary as we know it with the work of the out-patient department of a hospital, and some have branched out into lines of research that bring them into close touch with the political economist and the social worker. A study of the system is of interest to us just now because it presents paths the British medical profession may possibly follow in the near future..

A dispensary is defined by Dr. S. S. Goldwater, the Commissioner of Health of New York City, as "a place to which ambulant patients may come for gratuitous or semi-gratuitous examination, advice, and treatment." Such a definition will include many institutions varying widely in their organisation and methods of work. The regulations governing them vary in the different States. In New York dispensaries have to obtain a licence from the State Board of Charities. Applicants for a licence have to give proof of a benevolent interest in the work as attested at least by the willingness to spend enough money to carry on the work in a proper way. Dr. Goldwater considers that more stringent regulations are required, and says that not a few licensees fail to carry on their work in a satisfactory manner. The number of dispensaries is rapidly growing—in 1900 there were 100; in 1910, 574; and in 1915 more than 700. In 1910 nearly two million sick persons were treated in institutions in the United States, of which number dispensary patients formed the greater part. In New York alone it is estimated that nearly one million persons look to dispensaries for treatment. There are both general and special dispensaries. The special dispensaries have been formed chiefly in connection with the treatment of tuberculosis and with the prevention of infantile mortality. Many of the large mercantile companies have established small dispensaries to deal with the emergency treatment of accidents and with the more trivial ailments of their employees. The New York City department of health has gone a step further. It has established a dispensary which deals not only with the sick but examines periodically all employees. The department hopes by this system to prevent illness and detect insidious chronic diseases before irremediable harm is done. Many of the general dispensaries are attached to hospitals, but most of them are run independently. The medical staff is in most cases unpaid, but in some cases it is paid. The dispensary attached to a hospital has obvious advantages. A patient at a hospital dispensary found to be in need of indoor treatment can be admitted promptly to hospital, and the dispensary staff can follow the patient to the wards. Patients can thus be handled from start to finish by one man or by a group of men working in harmony. Similarly, a patient in the hospital can be transferred to the dependent dispensary for after-care.

Many of the dispensaries have a social service department which deals with the patient's economic position and his environment from the point of view of their effects upon his health. The Boston dispensary, which, except as regards its children's department, is unconnected with any hospital, was the first to take up this work, and has carried it farthest. The work originated in the feeling that the failure of a patient to return for needed treatment was discreditable to the dispensary. A system was instituted to follow up such patients and to find out why they did not come again. Now the dispensary regularly sends nurses and social workers to the homes of the patients to collect facts as to their surroundings, their habits, and their social and occupational history. It is plain that such investigations, systematically and intelligently carried out and sifted, may lead to the discovery of facts bearing directly upon the causation and prevention of disease.

The large dispensaries have a specialist and a general staff, and admit medical students to their clinics. Their medical work linked up with their social service work forms a kind of "team work" that, with a few exceptions, is not to be found in England. At present the dispensaries are not as a rule available for patients who are self-supporting, but attempts are being made to extend their services to such patients. Such dispensaries, staffed by men practising medicine in their neighbourhood, and linked up with voluntary or municipal hospitals, would go far towards the realisation of the scheme of medical reform outlined by Sir Bertrand Dawson in his Cavendish lecture this year.

